# Porokeratotic eccrine ostial and dermal duct nevus: a unique case treated with CO_2_ laser

**DOI:** 10.1002/ccr3.846

**Published:** 2017-03-31

**Authors:** John Zade, Abdulhadi Jfri, Adam Nabatian, Abdullah Alajaji, Lauren Geller, Hooman Khorasani

**Affiliations:** ^1^ Department of DermatologyMount Sinai School of MedicineNew York CityNew YorkUSA

**Keywords:** Eccrine hamartoma, porokeratotic adnexal ostial nevus, porokeratotic eccrine and hair follicle nevus, porokeratotic eccrine ostial and dermal duct nevus

## Abstract

Porokeratotic eccrine ostial and dermal duct nevus (PEODDN) is a rare eccrine hamartoma, with treatment generally being unsatisfactory. The unique features of PEODDN presented include bilateral and facial lesions, and extensive body involvement. Management with CO_2_ laser was successful, and follow‐up will be necessary to monitor for recurrent lesions.

## Introduction

Porokeratotic eccrine ostial and dermal ductal nevus (PEODDN) is a rare benign nevoid disorder characterized by cornoid lamella, which are a thin column of parakeratotic cells with a decreased or absent granular layer and vacuolated or dyskeratotic cells in the spinous layer that involve the acrosyringia 1. The condition was first named by Abell and Read in 1980 2 and first described a year earlier by Marsden et al. 3 as a comedo nevus of the palm 3. Recently, an effort has been made to re‐term the condition porokeratotic adnexal ostial nevus (PAON) to encompass PEODDN and porokeratotic eccrine and hair follicle nevus (PEHFN) as these conditions are very similar based on histopathology 4.

The classic presentation of PEODDN is multiple punctate or keratotic papules that appear on the extremities at birth or early in childhood 5. Although the distal extremities are the most common sites of occurrence and lesions are usually limited to this area, widespread involvement of the trunk, face, and proximal extremities has also been reported 6. The keratotic papules often coalesce into plaques and more progressed lesions usually distribute among Blaschko's lines 4, 5, 6, 7. Curative treatment options are limited, and the condition usually persists into adulthood 4.

## Case Presentation

An 18‐year‐old Hispanic male presented to the clinic with an itchy rash on the left half of the body. The rash was reported by the patient to be noticeable since birth and started on the left inner thigh as multiple papular lesions that slowly progressed over time to expand downward to the left foot. Papular lesions also appeared on the left side of his trunk as the patient grew older, which progressed to form linear plaques. Interestingly, the papular lesions on the patient's distal extremity were the last to appear. He never had a biopsy and was using anti‐itching products without significant improvement. The patient reported an aunt with a similar skin lesion who was never diagnosed. The patient was otherwise healthy and did not have any systemic complaints.

**Figure 1:**
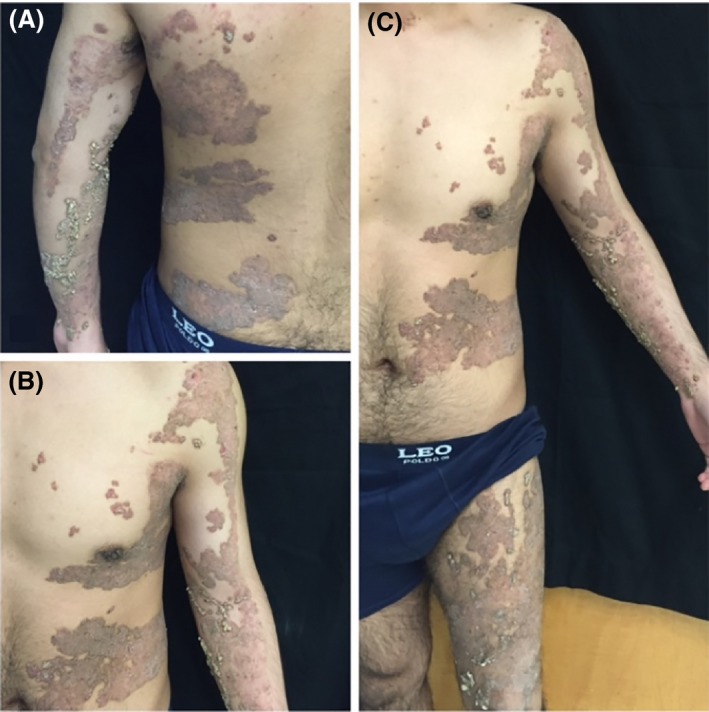
Multiple hyperkeratotic linear plaques on the left upper half of the body (A), involving the left side of the trunk and upper extremity (B), extending from the left thigh to the left foot (C).

**Figure 2:**
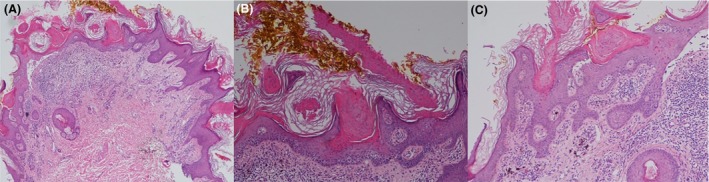
There is a verrucous epidermal hyperplasia with multiple diagonal columns of parakeratosis and scattered dermal lymphocytic infiltrates (A). At the base of the columns of parakeratosis are dyskeratotic keratinocytes, these features are consistent with cornoid lamellae (B, C).

On physical examination, there were multiple hyperkeratotic linear papules tending to coalesce into plaques on the left shoulder extending down to the left hand, left flank, left side of the back, left thigh, and down to the left foot (Fig. 1). There were also scattered multiple hyperkeratotic papules on the helix and a localized area on the right forehead. The histological examination showed multiple columns of cornoid lamella overlying acrosyringia, a distinctive feature of PEODDN (Fig. 2).

**Figure 3:**
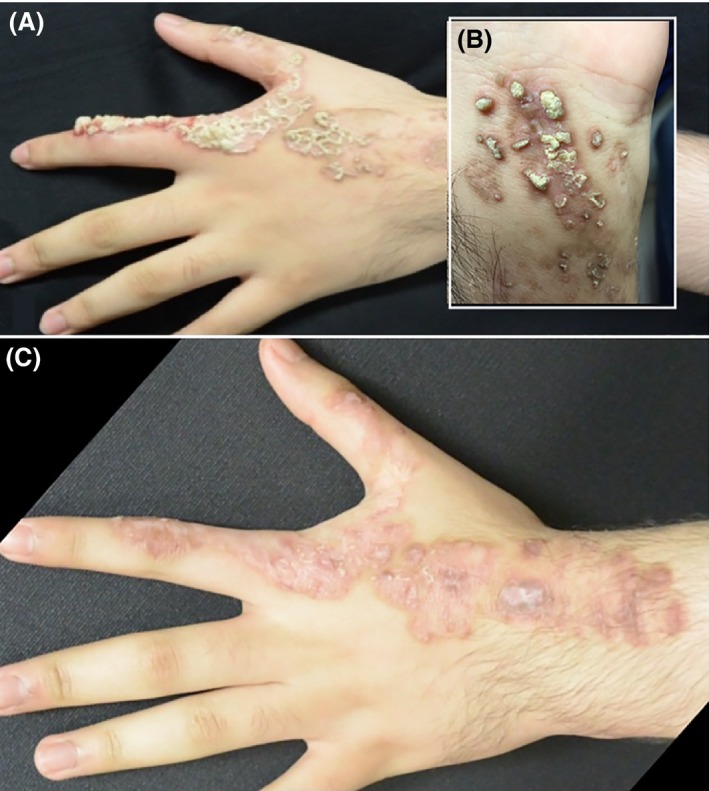
Left dorsum of the hand before fractional CO_2_ laser (A, B), Left dorsum of the hand after one session of fractional CO_2_ laser with 8 W (C).

The patient presented for follow‐up after being placed on tretinoin 0.1% cream, triamcinolone ointment, calcipotriene, and clobetasol propionate. After 2 months of treatment with these topical therapies, there was no observable improvement. The patient was then started on ultrapulse CO_2_ laser trail with a truspot handpiece on continuous mode with an energy setting of 8 W; two passes of curettage were performed between each pass. There was notable improvement after one treatment session. A second session of CO_2_ laser with the same settings was given after 1 month, which showed further improvement (Fig. 3).

## Discussion

Porokeratotic eccrine ostial and dermal duct nevus is thought to be an eccrine hamartoma that presents at birth and persists into adulthood. The condition is very rare and has a relatively equal occurrence in both genders 5. Although the pathogenesis of this disorder is unclear, it is believed that genetic mosaicism may play a role due to the involvement of Blaschko's lines 4. A study by Easton et al. 8 showed that PEODDN may be a mosaic form of keratitis ichthyosis deafness (KID) syndrome due to a somatic mutation in GJB2, which codes for the gap junction protein connexin 26 8.

Lesions for PEODDN are usually punctate pits or pitted papules in a linear pattern that are asymptomatic or mildly pruritic; these lesions are predominately located on the palms and soles 5. Lesions on other parts of the body frequently consist of multiple verrucous, keratotic, brown to flesh colored papules that may coalesce into linear plaques 5. Although long‐term involution has been reported in the literature 9, most lesions are persistent and some may progress in size. Lesions are thought to be benign; however, transformation of a lesion into Bowen's disease has been reported 10. In regard to distribution, of the 39 cases reviewed by Goddard et al. 4, 82% of them did not have bilateral involvement and only 33% had widespread involvement. The differential diagnosis for this condition includes epidermal nevi, including linear and verrucous epidermal nevus (ILVEN), linear psoriasis, linear Darier's disease, and forms of ichthyosis with a Blaschkoid distribution, such as Conradi syndrome and CHILD syndrome. Other medical conditions have been associated with PEODDN, including but not limited to hyperthyroidism, sensory polyneuropathy, deafness, developmental delay, and squamous cell carcinoma 11.

Our patient differs from many reported in the literature due to the extensive body involvement and paradoxical disease progression. Most cases of PEODDN start as spiny papules on the distal extremity and may progress to involve the proximal limb and trunk in the form of papules or coalescing linear plaques. In our case, the patient reported the initial lesion as papules on the inner thigh, followed by papules on the trunk and lastly appearing were the spiny papules on the distal extremity. The extensive involvement of the patient's left half of the body as well as the bilateral lesions on the face is also a rare finding in this disorder. In a review of literature by Goddard et al. 4, 41 previously reported PEODDN cases were analyzed and only eight were bilateral (19.5%) and only two reported cases involved the helical rims 4, 6.

Treatment options are generally limited and are not very successful, especially with larger lesions 4. Attempted treatments in the literature with disappointing results have included topical corticosteroids, calcipotriol ointment, phototherapy, and cryotherapy 4. Surgical excision may be a treatment choice for smaller and localized lesions; however, recurrence may still occur 4, 12. Relatively successful treatment for larger lesions has been observed with CO_2_ laser 13, 14 and combined erbium/CO_2_ laser 15. Multiple treatments and follow‐up after laser therapy may be required as recurrence of treated lesions has been reported 14.

## Conclusions/Future Directions

In conclusion, we report a case of symptomatic PEODDN with distinguishing clinical characteristics and widespread involvement. Due to the rarity of the condition, all cases of PEODDN should continue to be reported in order to further our understanding and management of the condition. Our case was unique in that the lesions were bilateral, extensive, and involved the face and helical rims. The progression of the lesions was also notable, as papules appeared on the torso and proximal extremity prior to involvement of the distal extremities. Treatment of our patient with CO_2_ laser and curettage had a successful outcome, and future follow‐up will be necessary to observe for recurrence.

## Conflict of Interests

The authors have no conflict of interests, financial disclosures, or professional relationships to disclose.

## Authorship

JZ: first author; responsible for the introduction, discussion, and paper submission. AJ: responsible for the case presentation and review. AN: responsible for the clinical message, introduction, and review. AA: responsible for the case presentation and review. LG: responsible for the differential diagnosis, discussion, and conclusions. HK: principal investigator; responsible for the methods, revisions, discussion, and review.
